# The Use of the Cancellation Technique to Quantify the Hermann Grid Illusion

**DOI:** 10.1371/journal.pone.0000265

**Published:** 2007-02-28

**Authors:** Piers D.L. Howe, Margaret S. Livingstone

**Affiliations:** Department of Neurobiology, Harvard Medical School, Boston, Massachusetts, United States of America; Indiana University, United States of America

## Abstract

When observers view a grid of mid-gray lines superimposed on a black background, they report seeing illusory dark gray smudges at the grid intersections, an effect known as the Hermann grid illusion. The strength of the illusion is often measured using the cancellation technique: A white disk is placed over one of these intersections and the luminance of the disk is reduced until the disk disappears. Its luminance at this point, i.e., the disk's detection threshold, is taken to be a measure of the strength of the illusion. Our experiments showed that some distortions of the Hermann grid, which were sufficient to completely disrupt the illusion, did not reduce the disk's detection threshold. This showed that the cancellation technique is not a valid method for measuring the strength of the Hermann grid illusion. Those studies that attempted to use this technique inadvertently studied a different effect known as the blanking phenomenon. We conclude by presenting an explanation for the latter effect.

## Introduction

**Figure 1:**
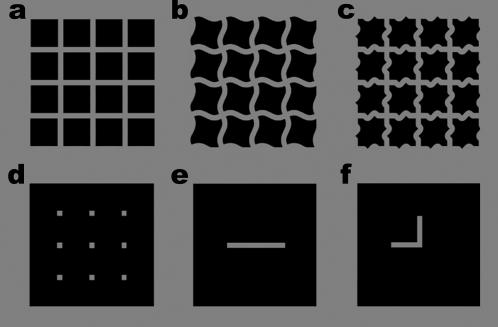
The six displays used in the experiments. Display (a) is the Hermann grid display [1]. Most observers see illusory dark gray smudges at the grid intersections. The illusion is strongest when viewed on a computer monitor. Displays (b) and (c) are based on displays that were presented at the European Conference on Visual Perception (Geier, Sera, Bernath, 2005, *Perception* 34, supplement 54).

Figure 1a shows an example of the Hermann grid illusion [1]. It consists of a grid of mid-gray lines on a black background. At the intersections of the lines illusory dark gray smudges are seen. The strength of the illusion is often measured using the cancellation technique, according to which a white disk is placed on an intersection of the grid, and the luminance of the disk is decreased until the disk disappears. The disk's luminance at the point of disappearance is used as a measure of the apparent luminance of the dark gray smudges [2]–[5].

In this paper we will show that some distortions of the Hermann grid display, which are sufficient to cause the dark gray smudges to entirely disappear, do not decrease the detection threshold of the disk. This means that the disk's detection threshold cannot be used as a measure of the apparent luminance of the dark gray smudges, so the cancellation technique is not a valid method for measuring the strength of the Hermann grid illusion.

## Materials and Methods

Four observers were used. All were males between the ages of 20 and 30 years. One was an author of this paper and the other three were unaware of the purpose of the research. All were experienced psychophysical observers and had either normal or corrected-to-normal visual acuity. All procedures were approved by the Harvard University Institutional Review Board for the use of human subjects.

Stimuli were displayed on a CRT monitor with 1024×768 pixels and an 85 Hertz refresh rate. The monitor was calibrated using a Pritchard® 1980A photometer. Using a combined head and chin rest, observers viewed the monitor from a distance of 63.5 cm. At this distance each pixel subtended approximately 1.4 arc minutes.

The Hermann grid and two variations (Figure 1a–c) were presented in turn at the center of the monitor. Each grid was 6.1° wide by 5.9° high. The luminances of the white, gray, and black regions were 145 cd/m^2^, 56.3 cd/m^2^ and 6.18 cd/m^2^ respectively. Observers freely viewed each display for as long as they needed and were required to indicate if they saw any dark gray smudges at the grid intersections. All observers reported that they readily saw dark gray smudges at the intersections of the Hermann grid display but, consistent with a report from the European Conference on Visual Perception (Geier, Sera, Bernath, 2004, *Perception* 33, supplement 53), the observers did not see any dark gray smudges at the intersections of the other two displays.

Each observer started the next experiment by adapting to the mid-gray background for 30 seconds. A white 1.2°×1.2° fixation cross was then presented at the center of the monitor. While the observer maintained fixation on the cross he pressed a key to start the trial. Five hundred milliseconds later a disk of diameter 0.27° was flashed randomly either to the left or to the right of the cross at a distance of 4.3° from the cross. As is conventional [6], [7], the disk was presented briefly (141 ms) to prevent the observer changing fixation. The timing of the display was confirmed with an oscilloscope. The observer was required to indicate on which side of the fixation cross the disk had appeared. A staircase procedure was used to estimate the detection threshold of the disk. Initially the disk was white, but after two correct responses its luminance was decreased. Conversely, after one incorrect response the luminance of the disk was increased. The luminance of the disk was constrained to be always greater than the luminance of the gray background (56.3 cd/m^2^) but less than the maximum luminance of the monitor (145 cd/m^2^). The size of the descending step was always 0.5488 of the size of the ascending step. This ratio was chosen because it has been shown to result in highly stable measurements [8]. Following this procedure, the staircase converged on the luminance value at which there was an 80.35% probability of the observer detecting the disk. The staircase was terminated after nine reversals in direction, and the luminance values of the last six reversals were averaged to produce the staircase's estimate of the detection threshold. For each observer the staircase procedure was run ten times and the results averaged. For the duration of the staircase, the size of the ascending step was kept as a constant fraction of the disk luminance because this produces more accurate results [8]. To minimize the effect of one staircase on another, each staircase used a different step size.

## Results

### Experiment 1 – Detection thresholds for an isolated disk

**Figure 2:**
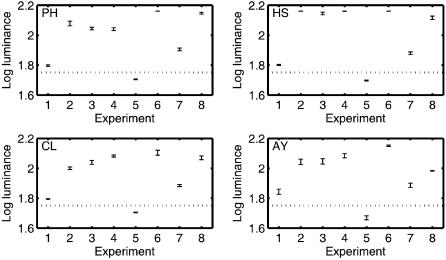
The experimental results. For each experiment, the plots show the luminance required for the target disk to be detected 80.35% of the time. The error bars represent the standard error of the mean. For some points the errors are so small that the error bars corresponding to those points collapse into horizontal lines. The horizontal dotted line represents the luminance of the gray background.

The results for this and the other experiments are shown in Figure 2. Depending on the observer, the disk was detected when it had a luminance 4–9% greater than the luminance of the background.

### Experiment 2 – Detection thresholds when the disk was embedded in Figure 1a

We measured how much the detection thresholds of the disk were increased by placing the Hermann grid shown in Figure 1a around it. To do this we used the same procedure as before, except in this experiment we placed a Hermann grid on either side of the fixation cross. Each Hermann grid was the same size as the ones used previously, and the central intersection of each grid was 4.3° degrees from the fixation cross. This ensured that regardless on which side the gray disk appeared, the gray disk was always centered on the central intersection of that side's Hermann grid. The width of the lines of the grid equaled the diameter of the gray disk. Following the same procedure used in the first experiment, the observers had to indicate whether the gray disk appeared to the left or to the right of the fixation cross. The means for each observer in each experiment were calculated and by performing a paired two-tailed t-test on these means we found that the thresholds in Experiment 2 were significantly greater than the thresholds in Experiment 1: t(3) = 4.45, p<0.02.

### Experiments 3 and 4 - Detection thresholds when the disk was embedded in Figures 1b and 1c

If the lines in the Hermann grid are bent so that they form a sine wave (Figure 1b) or a series of knots (Figure 1c), then the illusion completely disappears, as was confirmed by our four subjects in the initial screening. If the disk detection thresholds are a valid measure of the strength of the Hermann grid illusion experienced by each observer, then these manipulations should significantly reduce them. To test this we replaced the Hermann grid used in Experiment 2 with the sine wave grid and a knotted grid. Contrary to this prediction, and consistent with a report presented at the Vision Sciences Society (Levine, M. W., & McAnany, J. J., 2006, *Journal of Vision, 6*(6):902), the detection thresholds of Experiment 3 and Experiment 4 were not significantly less than those of Experiment 2: t(3) = 0.49, p = 0.65 and t(3) = 0.70, p = 0.54 respectively. These results show that the disk detection thresholds a not a valid measure of the strength of the Hermann grid illusion.

### Experiment 5 - Detection thresholds for a dark disk

It could be argued that observers did not see any dark gray smudges in Figures 1b and 1c because in these displays the smudges were just below the detection threshold. In Experiment 5 we measured the detection thresholds of a dark spot on a gray background. This should be equal to the limit on how dark the hypothesized dark gray smudges in Figures 1b and 1c could be given that they were not detected. If these non-visible dark gray smudges were the reason why the detection thresholds in Experiments 3 and 4 were greater than those in Experiment 1 then, for each observer, the difference in the detection thresholds between Experiment 1 and either Experiment 3 or 4 should be less than or equal to the detection threshold measured in Experiment 5. Contrary to this prediction, the detection thresholds of Experiment 5 were less than the difference between the detection thresholds of Experiments 3 and 1, t(3) = 7.70, p<0.01, and also less than the difference between the detection thresholds of Experiments 4 and 1, t(3) = 7.20, p<0.01.

### Experiment 6 - Detection thresholds when the disk was embedded in Figure 1d

Experiments 3 and 4 showed that manipulations which were sufficient to destroy the Hermann grid illusion did not reduce the disk detection thresholds. In this experiment we made a further attempt to reduce the disk detection thresholds. Since Experiments 3 and 4 showed that manipulations of the lines joining the intersections had little effect on the disk detection thresholds, we decided to remove these lines completely leaving only the grid intersections (Figure 1d). Despite this drastic manipulation, the disk detection thresholds in this experiment were still much greater than those in Experiment 1: t(3) = 11.5, p<0.01.

### Experiments 7 and 8 - Detection thresholds when the disk was embedded in Figure 1e and 1f

Experiments 7 and 8 were designed to investigate how collinearity affects the disk detection thresholds. Figures 1e and 1f both consist of a single gray line on a black background and were used in Experiments 7 and 8 respectively. In Experiment 7 the gray line was positioned so that the gray disk coincided with its center, and in Experiment 8 the line was positioned so that the gray disk coincided with the bend in the line. The thresholds in Experiment 8 were significantly greater than the thresholds in Experiment 7: t(3) = 4.57, p = 0.02. This result shows that the disk detection thresholds are increased when the abutting lines are not collinear.

## Discussion

In the Hermann grid display, illusory dark gray smudges are seen at the intersections of a grid. The strength of this illusion has often been measured using the cancellation technique, according to which a white disk is placed at an intersection and the luminance of this disk is reduced until the disk disappears. It is assumed that this occurs when the combination of the disk's luminance and the apparent luminance of the illusory dark gray smudge at the disk's location is equal to the mid-gray background. The disk's detection threshold is consequently taken to be a measure of the strength of the Hermann grid illusion. Experiments 3 and 4 showed that manipulations of the Hermann grid which were sufficient to destroy the illusion did not decrease the disk detection thresholds. This demonstrated that the cancellation technique is not a valid method for measuring the strength of the Hermann grid illusion.

Since the high disk detection thresholds measured when the disk was surrounded by a Hermann grid cannot be explained in terms of the Hermann grid illusion, it constitutes a distinct effect, worth studying for its own sake. It is typically referred to as the blanking phenomenon [6], [7]. Those studies that attempted to use the cancellation technique to measure the strength of the Hermann grid illusion [2]–[5] inadvertently studied the blanking phenomenon.

### The center-surround explanation of the blanking phenomenon

**Figure 3:**
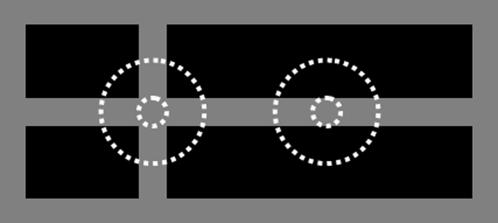
The receptive fields of two cells. For each cell, the inner circle and the area between the two circles represent the regions where stimulation by a light source respectively leads to excitation and inhibition of the cell. Please see the text for further details.

McAnany and Levine [6] proposed an explanation for the blanking phenomenon. They suggested a two-stage account, with one stage occurring before and the other stage after the site of binocular fusion. Unfortunately, they were unable to describe the second stage, so our discussion of their theory has to be confined to the first stage. This stage utilized the on-center off-surround receptive fields of the retina as shown by Figure 3. In this figure the receptive field on the left is centered on an intersection, whereas the one on the right is centered on a line. The surround of the receptive field on the left receives more excitation than the one on the right, so the cell corresponding to the receptive field on the left experiences more inhibition than the cell corresponding to receptive field on the right. The difference in inhibition could explain why the blanking phenomenon is strongest when the light gray disk is centered on an intersection rather than on a line.

This center-surround theory can readily explain why the detection thresholds are approximately equal for Experiments 2–4. In the displays used in these experiments (Figures 1a–1c) the amount of gray and black surrounding the intersections were identical, so the mean luminance of these surrounds must also have been identical. Since the theory postulates that it is the mean luminance of the surround that determines the detection threshold of the disk, this would explain why the blanking phenomenon was approximately equal for all three figures.

However, this theory cannot explain why the detection thresholds measured in Experiment 6 were much larger than those measured in Experiment 1. In Experiment 6 the display consisted of gray squares on a black background (Figure 1d). The disk was always placed on the center square of either the left or right display. When a cell's receptive field is centered on one of these squares, its surround is not stimulated, so the cell receives little inhibition, which should cause it to have a low detection threshold. Conversely, in Experiment 1 the background was a uniform gray, so the surround of a cell's receptive field centered on either the left or right display should receive substantial excitation causing the cell to be strongly inhibited and therefore have a high detection threshold. The center-surround theory therefore predicts the detection thresholds to be higher in Experiment 1 than in Experiment 6, the opposite of what was actually observed.

The center-surround theory also cannot explain the results of Experiments 7 and 8. In each case the disk is surrounded by the same amount of gray and black, so the center-surround theory would predict that the thresholds for these two experiments should be the same. Contrary to this prediction the thresholds in Experiment 8 were considerably higher than those in Experiment 7.

### A new explanation for the blanking phenomenon

In Experiment 6 the observer had to determine whether the light gray disk appeared in the left or right display. In both displays everything but the intersections of the Hermann grid had been removed leaving a grid of nine gray squares. The disk was always located over the center square of either the left or right display and had the same diameter as the width of the square on which it was centered. Observers reported that the disk's outline could not be distinguished from the outline of the square on which it was located. Consequently, placing the disk on a square increased the brightness of the square which in turn increased the contrast between the square and the background. Observers reported that they detected the gray disk by comparing the center squares of the left and right displays and then by assuming that the one with the higher contrast was the one with the disk superimposed on it. According to Weber's law, the difference in contrast between the two squares that is just detectable is proportional to the mean contrast of the squares. Because the contrast between the squares and the background was large Weber's law correctly predicts that the disk detection thresholds should have been large. Similarly, in Experiments 2–4 the contrast between the regions where the disk could be located and the background was large, which explains why the thresholds measured in these experiments were also large.

Weber's law cannot explain why the detection thresholds measured in Experiment 7 were lower than those in Experiment 8. In these two experiments the amount of black and gray in the area surrounding the disk was the same, so Weber's law incorrectly predicts that the thresholds of these two experiments should also have been the same. To explain why they were not we need to consider spatial facilitation.

In certain circumstances an item is easier to detect if it is flanked by two other items that are collinear with it, a phenomenon known as spatial facilitation. For a review see [9]. In Experiment 7, the disk was positioned on the center of a straight line, whereas in Experiment 8 it was positioned on the bend of a line. Collinear facilitation can therefore explain why the detection thresholds were less in Experiment 7 than in Experiment 8.

One might wonder why collinear facilitation did not cause the detection thresholds measured in Experiment 2 to be small. We suggest that it did not because in that experiment the disk was located at the intersection of two orthogonal lines. Facilitation did not occur because these lines inhibited each other. Inhibition of facilitation has been observed in a similar circumstance [10].

The above constitutes an outline of an explanation of the blanking phenomenon. It still needs to be converted into a precise mathematical format so that it can make definite, quantitative predictions. To do this would require numerous additional experiments to be conducted. For example, although it has been shown that the blanking phenomenon continues to exist even when the grid is reduced to a single intersection [7], preliminary results suggest that the illusion increases if the grid is made larger. If true then this would need to be described fully before a complete account of the blanking phenomenon could be given.
